# Screening for Cough Variant Asthma Using the TabPFN Model on Routine Laboratory Data in a Multicenter Cohort: Prospective Validation Study

**DOI:** 10.2196/92211

**Published:** 2026-10-23

**Authors:** Li-jie Yang, Shi-ning Wang, Xiu Shi, Ni-shan Deng, Miao-juan Zhu, Yun-fan Wang, Han-xiang Nie

**Affiliations:** 1 Department of Respiratory and Critical Care Medicine Renmin Hospital of Wuhan University Wuhan, Hubei China; 2 Department of Gastroenterology Renmin Hospital of Wuhan University Wuhan, Hubei China; 3 Hubei Key Laboratory of Metabolic and Chronic Diseases Renmin Hospital of Wuhan University Wuhan, Hubei China

**Keywords:** cough variant asthma, machine learning, TabPFN, Tabular Prior-Data Fitted Network, routine laboratory data, prospective validation

## Abstract

**Background:**

Cough variant asthma (CVA) is a prevalent but underdiagnosed subtype of chronic cough, largely due to the limited accessibility of pulmonary function tests in primary care.

**Objective:**

This study aimed to develop and prospectively validate an interpretable machine learning model using routine laboratory data to facilitate accessible CVA screening.

**Methods:**

We conducted a multicenter study combining retrospective model development and prospective validation using data from January 2021 to August 2025. The study included 3 sets—a retrospective development set (n=533; 428 training and 105 internal testing) and 2 prospective validation sets (internal validation: n=59 and external validation: n=78). In total, 54 routine biochemical, coagulation, and hematological markers were analyzed. A Tabular Prior-Data Fitted Network (TabPFN) model was developed and benchmarked against extreme gradient boosting (XGBoost). Performance was evaluated via area under the curve (AUC), accuracy, and specificity. Shapley Additive Explanations (SHAP) and decision curve analysis (DCA) were used to assess interpretability and clinical utility.

**Results:**

In external validation, TabPFN achieved an AUC of 0.858 (95% CI 0.763-0.943), accuracy of 79.5%, sensitivity of 71.4%, and specificity of 86%. XGBoost achieved a higher external AUC of 0.912 (95% CI 0.842-0.982) and sensitivity of 91.4%, but lower accuracy (75.6%) and specificity (62.8%) at the fixed 0.5 probability threshold. TabPFN retained higher AUCs than XGBoost in internal testing (0.964 vs 0.890) and temporal validation (0.925 vs 0.846). DCA demonstrated that TabPFN provided higher net benefit across clinically relevant threshold probabilities, with the range varying across validation cohorts. SHAP analysis identified serum sodium (Na) and prothrombin time international normalized ratio as the leading predictors, followed jointly by mean corpuscular hemoglobin concentration and red cell distribution width-SD, which showed the same rounded mean absolute SHAP value at the reported 2-decimal precision, and elevated sodium levels (>141 mmol/L) were associated with increased predicted risk.

**Conclusions:**

The TabPFN model demonstrated robust prospective performance as an accessible screening tool for CVA, with balanced threshold-dependent classification performance across validation cohorts. Although TabPFN showed higher discrimination than XGBoost in internal and temporal validation, XGBoost achieved a higher AUC in the external cohort, indicating that neither model uniformly outperformed the other across all performance dimensions.

## Introduction

Chronic cough, defined as a cough lasting >8 weeks, affects nearly 10% of the global population and is a leading reason for medical consultation [1]. Cough variant asthma (CVA), characterized solely or predominantly by coughing, accounts for 25%-42% of chronic cough cases worldwide [2]. Despite its prevalence, CVA is frequently underdiagnosed due to the absence of typical asthmatic signs like wheezing [3]. Current guidelines mandate objective evidence of variable expiratory airflow limitation for diagnosis, typically via bronchial hyperresponsiveness or bronchodilator reversibility testing [4,5]. However, these pulmonary function tests (PFTs) are significantly underused in primary care due to equipment costs and the need for technical expertise [6]. Particularly in resource-limited settings, the scarcity of spirometers creates a substantial “diagnostic barrier,” leading to high missed diagnosis rates [7,8]. Consequently, there is an urgent need for a more accessible screening tool.

Routine laboratory tests, a standardized component of electronic medical records, offer a promising alternative. Unlike specialized PFTs, these tests are widely available and cost-effective across all health care tiers [9]. While CVA is primarily an airway disease, it is driven by systemic type 2 inflammation and immune dysregulation, traces of which may be detectable in peripheral blood [10]. Previous studies have used single biomarkers, such as blood eosinophil counts, as proxies for airway inflammation [10]. However, single biomarkers often lack sensitivity. Traditional statistical methods fail to capture the complex, nonlinear interactions between multiple physiological parameters that collectively reflect the asthmatic status.

Machine learning (ML) excels at modeling these high-dimensional, nonlinear relationships in clinical data [11]. Although ML has shown promise in respiratory diseases [12,13], its application to CVA diagnosis using routine laboratories remains unexplored. Moreover, conventional “black-box” models often lack the interpretability necessary for clinical adoption [14]. To address the dual challenges of predictive performance on tabular medical data and model transparency, we used the Tabular Prior-Data Fitted Network (TabPFN). As a transformer-based foundation model, TabPFN uniquely integrates a Transformer architecture with a Bayesian framework. This enables state‑of‑the‑art predictions on small‑to‑medium‑sized tabular datasets without extensive training [15]. We further enhanced clinical interpretability using Shapley Additive Explanations (SHAP) to elucidate feature contributions [16] and decision curve analysis (DCA) to quantify net clinical benefit [17].

This study aimed to develop and validate an interpretable, TabPFN-based screening model for CVA using only routine laboratory data. Rather than proposing a novel algorithm, our core contribution lies in clinical translation. By applying TabPFN to widely available, low-cost laboratory data, we provide a practical screening tool that may mitigate the small-sample overfitting typical of traditional ML. We developed the model in a retrospective development cohort and assessed its robustness in prospective temporal and external validation cohorts. Our work provides a validated, low-cost framework to bridge the diagnostic gap for CVA in primary care settings.

## Methods

### Overview of the Analytical Pipeline

The overall methodology of this study was conducted in 5 sequential phases, as illustrated in Figure 1. First, patient data were retrospectively and prospectively collected from 2 institutions, and rigorous inclusion and exclusion criteria were applied to assemble the study cohorts. Second, the tabular data underwent strict preprocessing, including feature standardization and missing value management. Third, predictive models were developed. We systematically compared our primary TabPFN against a comprehensively optimized extreme gradient boosting (XGBoost) baseline. Fourth, the diagnostic performance of these models was rigorously evaluated across the training, internal testing, and 2 prospective validation cohorts. Finally, SHAP and DCA were applied to elucidate the biological relevance of the predictors and assess the real-world clinical utility of the final model.

**Figure 1:**
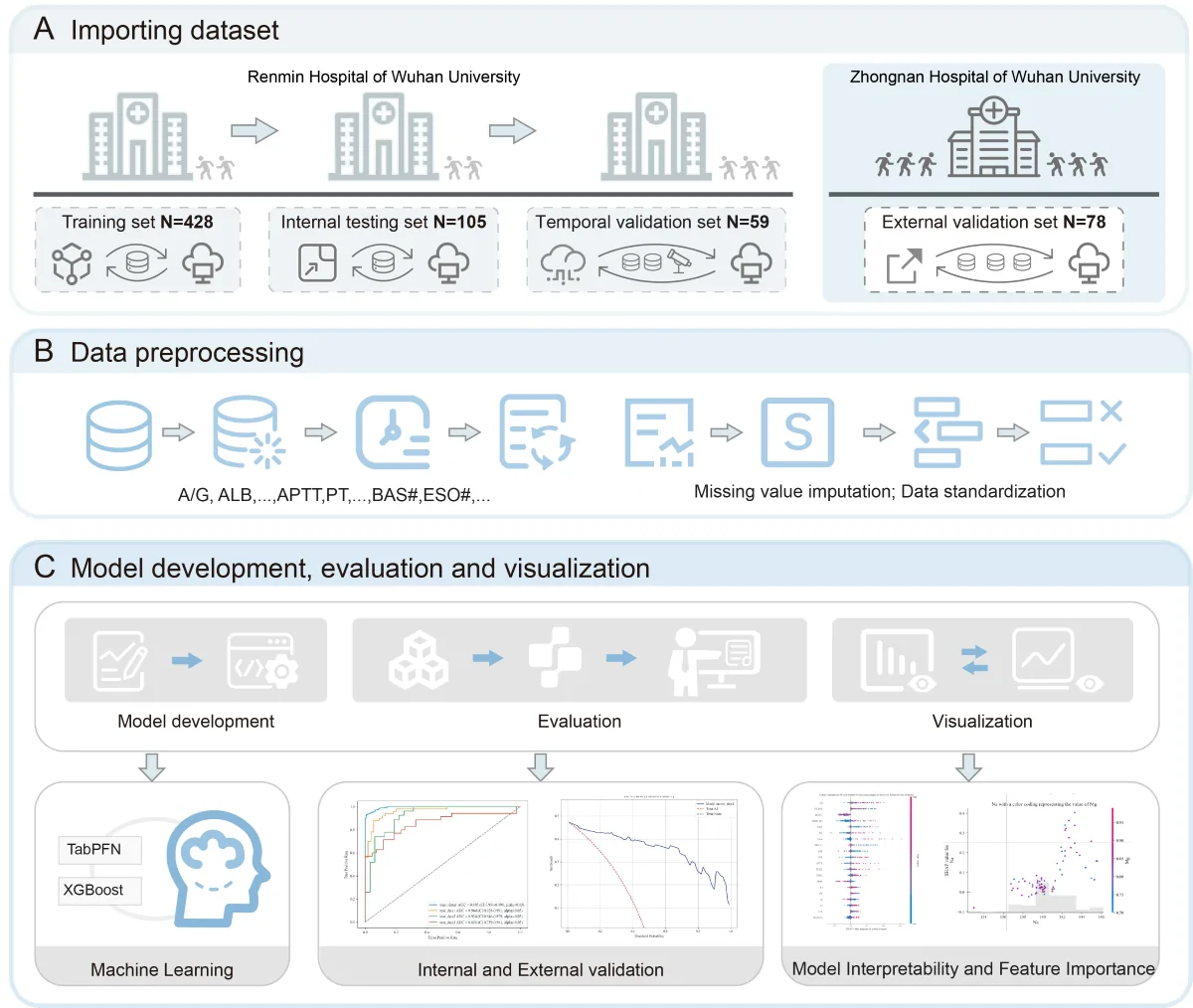
The overall framework of the study. (A) Data collection and set management: Patient data from two hospitals were partitioned into a training set (n=428), an internal testing set (n=105), a temporal validation set (n=59), and an external validation set (n=78). (B) Data preprocessing and feature extraction: Missing value imputation and feature standardization were performed. (C) Model development, evaluation and visualization: The TabPFN and XGBoost models were developed and evaluated using receiver operating characteristic curves and decision curve analysis, followed by SHAP analysis for feature interpretability. TabPFN, tabular prior-data fitted network; XGBoost, extreme gradient boosting; SHAP, Shapley Additive Explanations.

### Study Population

This study included patient data from 3 distinct cohorts to ensure rigorous development and validation of the model. The retrospective development sets were derived from Renmin Hospital of Wuhan University, covering the period from January 2021 to December 2024. We identified 533 eligible patients with a well‑balanced class distribution (n=258, 48.4% patients with CVA, and n=275, 51.6% patients with non‑CVA controls). These patients were randomly split at the patient level into a training set (n=428) and an internal testing set (n=105) in an approximate 8:2 ratio. The detailed class distribution for all cohorts is provided in Table S3 in Multimedia Appendix 1). To evaluate robustness and generalizability, we recruited 2 prospective validation sets between January 2025 and August 2025. The first was a temporal validation set (n=59) from the same institution, consisting of 27 (45.8%) CVA and 32 (54.2%) controls. The second was an external validation set (n=78) prospectively recruited from Zhongnan Hospital of Wuhan University, with 35 (44.9%) CVA and 43 (55.1%) controls. The detailed raw class distribution and baseline characteristics for all cohorts are provided in Table S3 in Multimedia Appendix 1).

The inclusion criteria were as follows: (1) patients aged 18 to 80 years; (2) primary complaint of chronic cough; (3) completion of at least 1 standard PFT (including bronchial provocation test or bronchial dilation test) with qualified results; and (4) availability of routine biochemical, coagulation, and complete blood count data obtained during the initial visit and sufficient to satisfy the prespecified missingness criterion. The exclusion criteria were: (1) failure to discontinue β2-agonists as required before the bronchial dilation test; (2) pregnancy; (3) acute kidney injury or chronic kidney disease; (4) malignant tumors; and (5) incomplete key clinical data due to transfer or other administrative reasons.

Patients were divided into 2 groups based on PFT results and final diagnosis—case group (CVA group) and control group (non-CVA group). In the case group, patients who met the following clinical criteria (cough as the sole or primary symptom) and objective criteria were included: (1) a positive bronchial dilation test (an increase in FEV_1_≥12% and an absolute increase ≥200 mL after bronchodilator inhalation), or (2) a positive bronchial provocation test (eg, a ≥20% decrease in FEV₁ with methacholine), or (3) a peak expiratory flow variability >10%, and (4) were ultimately diagnosed with “cough variant asthma.” The control group (non-CVA chronic cough group) included patients with negative pulmonary function test results who received a definitive diagnosis of an alternative cause of chronic cough (such as upper airway cough syndrome or gastroesophageal reflux-related cough) following systematic specialist evaluation.

### Data Collection

Laboratory test data and demographic characteristics were collected from the electronic medical records of patients with chronic cough at Renmin Hospital of Wuhan University and Zhongnan Hospital of Wuhan University. Data for the retrospective development cohort were retrospectively extracted, whereas data for the temporal and external validation cohorts were prospectively collected. To minimize potential bias, features with more than 60% missing values were excluded from subsequent analyses. A total of 54 features were ultimately used to develop the prediction models. These included following 25 biochemical indicators: albumin-to-globulin ratio, albumin, alkaline phosphatase, alanine transaminase, aspartate transaminase, Calcium (Ca), Chloride (Cl), creatinine, direct bilirubin, estimated glomerular filtration rate, gamma-glutamyl transferase, globulin, glucose, high-density lipoprotein cholesterol, Potassium (K), lactate dehydrogenase, low-density lipoprotein cholesterol, Magnesium (Mg), Sodium (Na), total bilirubin, total carbon dioxide (CO_2_), triglyceride, total protein, urea, uric acid; 5 coagulation indicators: activated partial thromboplastin time (APTT), D-dimer, fibrinogen, prothrombin time (PT), prothrombin time international normalized ratio (PT-INR); and 24 complete blood cell count indicators: basophil count, basophil percentage, eosinophil count, eosinophil percentage, hemoglobin (Hb), hematocrit, lymphocyte count, lymphocyte percentage, mean corpuscular hemoglobin, mean corpuscular hemoglobin concentration (MCHC), mean corpuscular volume, monocyte count, monocyte percentage, mean platelet volume, neutrophil count, neutrophil percentage, plateletcrit, platelet distribution width, platelet-large cell ratio, platelet count, red blood cell count, red cell distribution width-coefficient of variation, red cell distribution width-standard deviation (RDW-SD), white blood cell count. The proportions of missing data for each variable are presented in Table S1 in Multimedia Appendix 1).

### Data Processing

#### Data Standardization

To eliminate bias arising from the varying magnitudes of different biochemical and hematological indicators, StandardScaler was applied to all numerical features. Crucially, to prevent data leakage, the scaler was fitted exclusively on the training dataset, and these learned parameters were then used to transform both the training and validation datasets. This process normalized the data to a standard normal distribution (mean 0, SD 1). Standardization was performed on the datasets for both the TabPFN and XGBoost models to ensure scale consistency. This facilitated stable convergence and comparable feature importance analysis.

#### Missing Value Imputation

Candidate laboratory variables were first screened for missingness, and features with more than 60% missing values were excluded. After feature-level screening, patients with more than 60% missing values across the retained laboratory features were excluded. Before feature standardization and model input, laboratory measurements were harmonized to common units, including conversion of MCHC to g/L. For XGBoost, missing values were imputed using k-nearest neighbors (KNN; K=15). The K value was selected based on the sensitivity analysis presented in Table S2 in Multimedia Appendix 1). Similar to the standardization process, the KNN imputer was fitted solely on the training data and subsequently applied to the validation sets, thereby avoiding data leakage. For TabPFN, no imputation was applied. After standardization, the dataset retaining the original missing values (not-a-number values) was input directly into the model, leveraging TabPFN’s native capability to handle missingness via in-context learning.

#### Model Development With TabPFN

We developed the primary predictive model using the TabPFN [15], a pretrained transformer foundation model specifically designed for classification tasks on small-to-medium tabular datasets. Structurally, TabPFN treats tabular classification as a sequence modeling task. Its core advantage lies in “in-context learning.” Instead of iteratively updating network weights via gradient descent like conventional algorithms, TabPFN accepts the training data (support set) and test data (query set) simultaneously. It generates predictions by approximating Bayesian inference in a single forward pass. This noniterative approach effectively guards against overfitting on small datasets and fundamentally eliminates the need for extensive hyperparameter tuning.

For our experiments, TabPFN was deployed using its robust default configuration, running an ensemble of 32 spatial feature rotations to maximize predictive stability [15]. Furthermore, post hoc isotonic regression was applied to the TabPFN outputs to improve probability calibration. The isotonic regression model was fitted exclusively on the training dataset, and the fitted calibrator was subsequently applied to the internal testing, temporal validation, and external validation cohorts. No data from the validation cohorts were used to fit the calibrator. Following calibration, a fixed probability cutoff of 0.5 was used to classify patients as CVA or non-CVA.

#### XGBoost Model Development

To evaluate TabPFN performance relative to an established baseline, XGBoost was selected as the benchmark. The model was trained using the fully preprocessed dataset (standardization and KNN imputation). Key XGBoost hyperparameters were optimized using grid search with stratified 5-fold cross-validation, with area under the receiver operating characteristic curve (AUROC) as the optimization metric. The complete hyperparameter search grid and final selected parameter values are provided in Multimedia Appendix 2. All procedures were performed with a fixed global random seed of 42 to ensure reproducibility. Stratified 5-fold cross-validation was used exclusively for hyperparameter selection. After the optimal hyperparameters were identified, the final XGBoost model was fitted and evaluated on each study cohort. The 95% CIs for the performance metrics reported in Table 1 were estimated using 2000 bootstrap resamples and represent sampling uncertainty rather than run-to-run variability. No post hoc recalibration procedure was applied to the XGBoost predictions. Threshold-dependent classification metrics for XGBoost were therefore calculated from the original predicted probabilities using the same fixed cutoff of 0.5.

**Table 1:** Predictive performance of the extreme gradient boosting model across training and validation sets.

Dataset	Accuracy (95% CI)	AUROC^a^ (95% CI)	Sensitivity (95% CI)	Specificity (95% CI)	Precision (95% CI)	*F*_1_-score (95% CI)
Training	98.4% (97%-99.3%)	0.999 (0.997-1.000)	99% (97.5%-100%)	97.7% (95.4%-100%)	97.6% (95.4%-99.3%)	0.983 (0.970-0.993)
Internal testing	78.1% (69.5%-85.7%)	0.890 (0.825-0.956)	82% (71.4%-92.2%)	74.5% (62.1%-85.2%)	74.5% (62.9-85.1%)	0.781 (0.689-0.862)
Temporal validation	72.9% (61%-83.1%)	0.846 (0.742-0.950)	88.9% (75.9%-100%)	59.4% (42.1%-75.9%)	64.9% (48.8%-79.4%)	0.750 (0.618-0.853)
External validation	75.6% (66.7%-84.6%)	0.912 (0.842-0.982)	91.4% (81.8%-100%)	62.8% (48.7%-76.3%)	66.7% (52.4%-79.1%)	0.771 (0.658-0.860)

^a^AUROC: area under the receiver operating characteristic curve.

#### Model Evaluation and Explanation

We assessed the diagnostic performance of both TabPFN and XGBoost across the training, internal testing, temporal validation, and external validation cohorts. Standard metrics included area under the curve (AUC), accuracy, sensitivity, specificity, precision (positive predictive value), and *F*_1_-score. For the performance estimates reported in Tables 1 and 2, 95% CIs were obtained using 2000 bootstrap resamples within each cohort. These bootstrap CIs were used to characterize sampling uncertainty around the estimated performance metrics. The probability cutoff for threshold-dependent classification metrics was fixed at 0.5.

**Table 2:** Predictive performance of the Tabular Prior-Data Fitted Network model across training and validation sets.

Dataset	Accuracy (95% CI)	AUROC^a^ (95% CI)	Sensitivity (95% CI)	Specificity (95% CI)	Precision (95% CI)	*F*_1_-score (95% CI)
Training	95.8% (93.7%-97.7%)	0.995 (0.991-0.998)	94.7% (91.2%-97.6%)	96.8% (94.2%-98.9%)	96.6% (93.9%-98.9%)	0.956 (0.933-0.975)
Internal testing	89.5% (83.8%-95.2%)	0.964 (0.931-0.992)	88.0% (78.6%-96.2%)	90.9% (83.3%-98%)	89.8% (81.2%-97.8%)	0.889 (0.820-0.951)
Temporal validation	83.1% (72.9%-91.5%)	0.925 (0.839-0.979)	77.8% (61.5%-92.9%)	87.5% (73.5%-97.1%)	84% (68.2%-96.2%)	0.808 (0.667-0.909)
External validation	79.5% (70.5%-88.5%)	0.858 (0.763-0.943)	71.4% (54.8%-86.2%)	86% (75.5%-95.6%)	80.6% (65.6%-93.3%)	0.758 (0.621-0.857)

^a^AUROC: area under the receiver operating characteristic curve.

To interpret the predictions of the TabPFN model, we used SHAP. Crucially, to ensure the clinical persuasiveness and validity of our interpretability analysis, the SHAP visualization experiments were conducted strictly on the unseen testing and validation sets, rather than the training set. This guarantees that the derived feature importances reflect the model’s true generalization capabilities on new patients rather than mere data memorization. Methodologically, we applied a model-agnostic SHAP explainer to calculate the marginal contribution of each feature toward the final prediction. We constructed SHAP summary plots to evaluate global feature importance and establish the directional impact (positive or negative) of specific biomarkers on the probability of CVA. Furthermore, local SHAP visualizations were used to decode instance-level reasoning, demonstrating how individual patient profiles interact with the model’s learned thresholds.

Calibration curves were generated for both TabPFN and XGBoost across the internal testing, temporal validation, and external validation cohorts to visually assess agreement between predicted probabilities and observed outcomes. As described above, TabPFN probabilities underwent post hoc isotonic calibration, whereas XGBoost probabilities were evaluated without post hoc recalibration. DCA was used to evaluate the clinical utility of both models.

#### Statistical Analysis

Statistical analyses and model development were performed using IBM SPSS (version 26.0), R (version 4.2.3; R Core Team), and Python (version 3.10.0; Python Software Foundation). Categorical variables were expressed as frequencies and percentages (n [%]) and compared using the chi-square test or Fisher exact test. The normality of continuous variables was assessed using the Kolmogorov-Smirnov and Shapiro-Wilk tests. Normally distributed data were reported as mean (SD) and compared using the Student *t* test. Nonnormally distributed data were presented as medians with IQRs and analyzed using the Mann-Whitney *U* test. A 2-sided *P* value of <.05 was considered statistically significant.

#### Sample Size Considerations

No a priori sample size calculation was performed for the prospective validation cohorts because their sizes were determined by consecutive recruitment during predefined study periods. We therefore did not use post hoc power calculations to characterize sample adequacy. Instead, the precision and uncertainty of model performance estimates are represented by the corresponding 95% CIs. The relatively limited sizes of the temporal and external validation cohorts should be considered when interpreting the precision and generalizability of the validation results.

### Ethical Considerations

The study protocol was reviewed and approved by the Ethics Committee of Renmin Hospital of Wuhan University (approval WDRY2026-K009). The study also received ethical recognition from the Ethics Committee of Zhongnan Hospital of Wuhan University. For the retrospective development cohort, the requirement for informed consent was waived by the Ethics Committee due to the observational nature of the study and the use of strictly anonymous and deidentified data, which ensured the privacy and confidentiality of all participants. For the prospective validation cohorts, written informed consent was obtained from all participants before their inclusion. Study participants did not receive any financial compensation for their involvement. Furthermore, no images or supplementary materials in this manuscript contain identifiable information of any individual participants. Written informed consent was waived for the retrospective set due to the observational nature of the study, which used routine clinical data.

## Results

### Patient Characteristics

The study design is illustrated in Figure 2. Baseline demographic and laboratory characteristics across the cohorts are summarized in Table S3 in Multimedia Appendix 1). In terms of demographics, the external validation cohort was younger than the retrospective development cohort (median age 44.50, IQR 34.25-55.00 y vs 50.00, IQR 38.00-62.00 y). Sex distribution was generally balanced across all 3 cohorts, with a slight female predominance.

**Figure 2:**
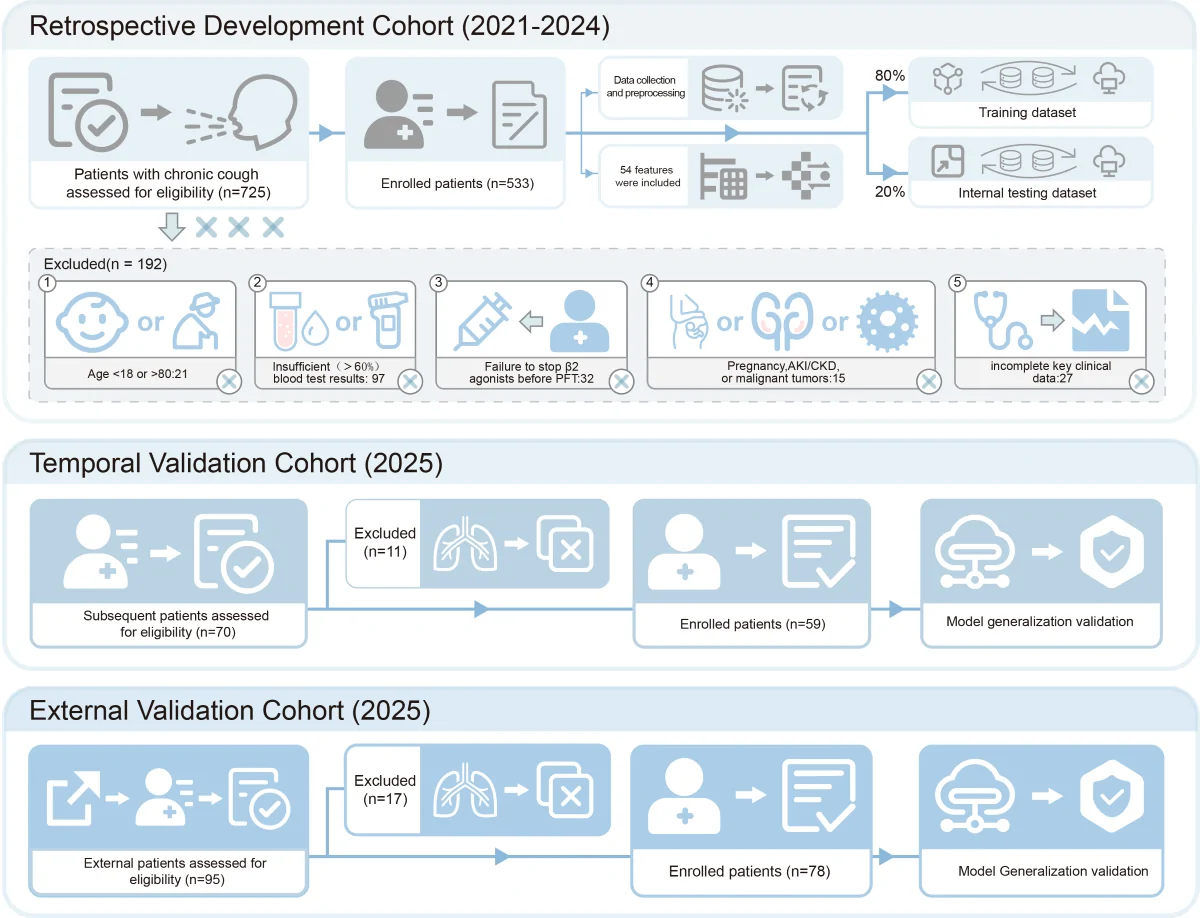
Flowchart of patient enrollment and cohort derivation. This flowchart details the screening, exclusion, and enrollment processes across 3 study cohorts. Retrospective development cohort (2021-2024): Among 725 initially assessed patients with chronic cough, 192 were excluded based on predefined clinical and data quality criteria (detailed in the figure). The remaining 533 eligible patients were randomly partitioned into a training dataset (80%) and an internal testing dataset (20%). Prospective validation cohorts (2025): To rigorously evaluate model generalization, 2 independent prospective cohorts were established—a temporal validation cohort (70 assessed, 11 excluded, and 59 enrolled) and an external validation cohort (95 assessed, 17 excluded, and 78 enrolled). CVA, cough variant asthma; PFT, pulmonary function test; AKI, acute kidney injury; CKD, chronic kidney disease.

### Predictive Performance and Clinical Utility of the TabPFN Model

The TabPFN model achieved an accuracy of 95.8% (95% CI 93.7%-97.7%) and an AUC of 0.995 (95% CI 0.991-0.998) in the training set. In the internal testing cohort, the model achieved an accuracy of 89.5% (95% CI 83.8-95.2), a sensitivity of 88% (95% CI 78.6-96.2), a specificity of 90.9% (95% CI 83.3-98.0), and an AUC of 0.964 (95% CI 0.931-0.992). Performance remained robust in the temporal validation cohort, with an accuracy of 83.1% (95% CI 72.9-91.5), a sensitivity of 77.8% (95% CI 61.5-92.9), a specificity of 87.5% (95% CI 73.5-97.1), and an AUC of 0.925 (95% CI 0.839-0.979). In the external validation cohort from Zhongnan Hospital of Wuhan University, the model achieved an accuracy of 79.5% (95% CI 70.5-88.5), a sensitivity of 71.4% (95% CI 54.8-86.2), a specificity of 86% (95% CI 75.5-95.6), and an AUC of 0.858 (95% CI 0.763-0.943; Table 2 and Figure 3A).

**Figure 3:**
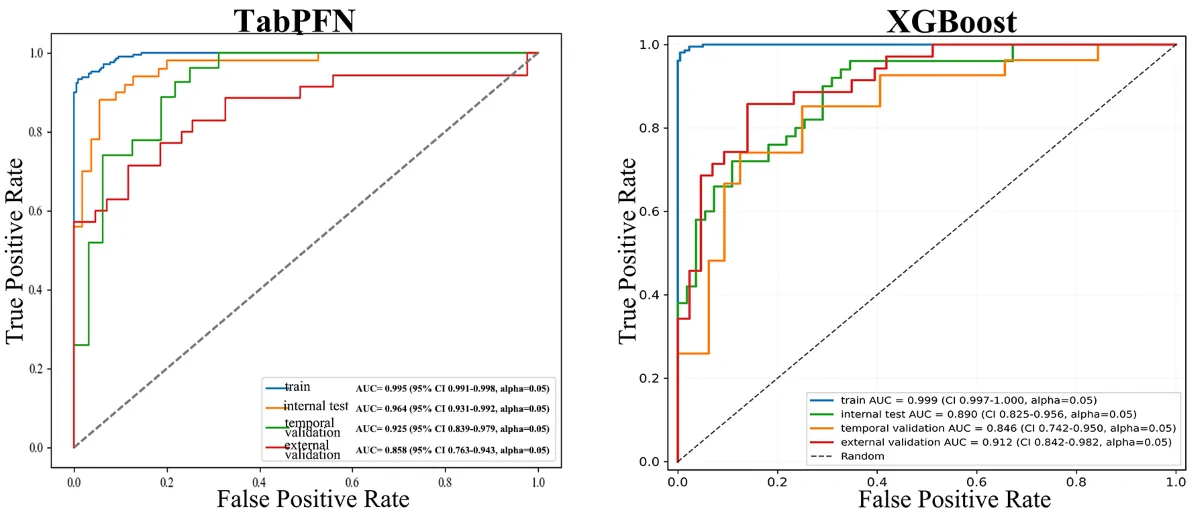
Comparison of discriminative performance between TabPFN and XGBoost models. (A) ROC curves of the TabPFN model across the training, internal testing, temporal validation, and external validation sets. (B) ROC curves of the XGBoost model across the same cohorts. AUC, area under the curve; ROC, receiver operating characteristic.

DCA was performed to assess the clinical utility of the model. Using the TabPFN model provided a higher net benefit than treating all patients or treating none in the temporal validation set. The model showed favorable net benefit across low-to-intermediate threshold probabilities (approximately 0.05 to 0.85). In the external validation set, positive net benefit extended to higher threshold probabilities, approaching 0.95. This indicates that the TabPFN model is useful for reducing unnecessary interventions while correctly identifying high-risk patients (Figure 4A-C).

**Figure 4:**
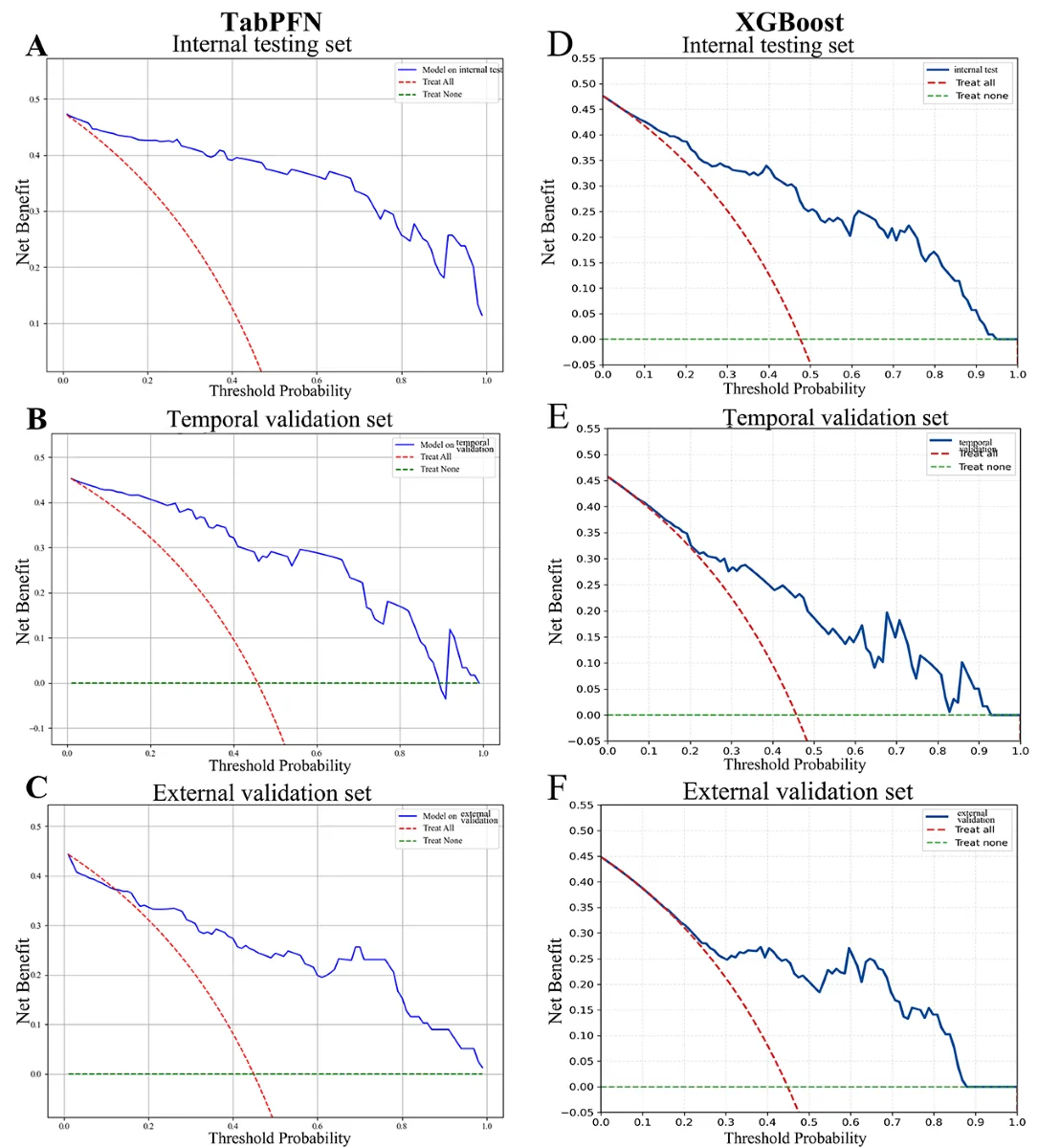
Comparative assessment of clinical utility using DCA. (A–C) DCA of the TabPFN model in the internal testing (A), temporal validation (B), and external validation (C) sets. (D–F) DCA of the XGBoost model in the internal testing (D), temporal validation (E), and external validation (F) sets. Interpretation: The threshold probability ranges showing positive net benefit varied across validation cohorts. The blue solid line represents the predictive model; the red dashed line represents the strategy of treating all patients; the green dashed line represents the strategy of treating no patients. CVA, cough variant asthma; DCA, decision curve analysis; TabPFN, tabular prior-data fitted network; XGBoost, extreme gradient boosting.

Calibration curves were additionally examined for both models (Figure S2 in Multimedia Appendix 1). The calibrated TabPFN probabilities showed generally good agreement with observed event frequencies across the validation cohorts. The unrecalibrated XGBoost curves showed deviations from the ideal calibration line in several probability ranges, with the pattern varying across cohorts. These plots were interpreted descriptively because post hoc isotonic calibration had been applied to TabPFN but not to XGBoost.

To further assess the model’s robustness, we conducted a subgroup analysis based on age, with a cutoff of 50 years representing the median of the retrospective cohort. The TabPFN model demonstrated consistent AUC-based discriminative performance across different age subgroups. In the external validation cohort, the model yielded an AUC of 0.869 (95% CI 0.739-0.971) in patients aged <50 years and 0.866 (95% CI 0.713-0.982) in those aged ≥50 years. In the temporal validation cohort, the corresponding AUCs were 0.909 (95% CI 0.797-0.991) and 0.945 (95% CI 0.846-1.000), respectively (Table S4 in Multimedia Appendix 1). Threshold-dependent performance showed greater variation across age strata. In the temporal validation cohort, sensitivity was 85.7% versus 69.2% and specificity was 77.8% versus 100% in patients aged <50 and ≥50 years, respectively. In the external validation cohort, the corresponding sensitivities were 78.9% and 62.5%, and the specificities were 79.3% and 100%. Given the relatively small subgroup sample sizes, these threshold-dependent estimates should be interpreted cautiously.

### Comparison With Benchmark XGBoost Model

TabPFN and XGBoost demonstrated different patterns of discrimination and threshold-dependent classification performance across the validation cohorts. XGBoost achieved near-perfect performance in the training cohort, with an accuracy of 98.4% (95% CI 97.0%-99.3%) and an AUC of 0.999 (95% CI 0.997-1.000). In the internal testing cohort, TabPFN achieved a higher AUC than XGBoost (0.964 vs 0.890), as was also observed in the temporal validation cohort (0.925 vs 0.846). In contrast, XGBoost achieved a numerically higher AUC in the external validation cohort than TabPFN (AUC=0.912, 95% CI 0.842-0.982 vs AUC=0.858, 95% CI 0.763-0.943), indicating that XGBoost retained strong threshold-independent discrimination in this cohort. At the fixed probability cutoff of 0.5, XGBoost showed higher external sensitivity than TabPFN (91.4% vs 71.4%) but lower specificity (62.8% vs 86.0%) and slightly lower accuracy (75.6% vs 79.5%). Thus, the relative performance of the 2 models differed according to the metric and validation cohort considered, rather than demonstrating uniform superiority of either model (Tables 1 and 2 and Figure 3).

DCA further demonstrated cohort-specific patterns of clinical net benefit for both models. TabPFN provided positive net benefit across broad ranges of threshold probabilities in the validation cohorts, whereas the magnitude and range of net benefit for XGBoost varied across cohorts (Figure 4A-4C). In contrast, the net benefit of the XGBoost model decreased in the external validation set compared with the internal testing set (Figure 4D-4F).

### Model Interpretability and Feature Importance

The SHAP summary dot plot (Figure 5A) illustrates the direction of feature effects. Elevated levels of Na and RDW-SD were associated with positive SHAP values, indicating an increased predicted risk. Conversely, PT‑INR showed an inverse relationship—lower values corresponded to higher risk estimates. Variables such as MCHC displayed nonlinear patterns. Global feature importance was ranked using the mean absolute SHAP values, presented in a bar plot (Figure 5B). As illustrated, Sodium (Na) ranked highest in relative importance, followed by PT-INR, while MCHC and RDW-SD showed comparable importance and both had a mean absolute SHAP value of 0.07 at the reported 2-decimal precision. Furthermore, the SHAP dependence plot for Na (Figure 5C) visualizes its interaction with Magnesium. It reveals a threshold effect—Na levels exceeding 141 mmol/L, which is the upper end of the normal range. While values above this level generally corresponded to a higher risk estimate, the wide variance in this range underscores a complex electrolyte interaction rather than a strict standalone clinical threshold.

**Figure 5:**
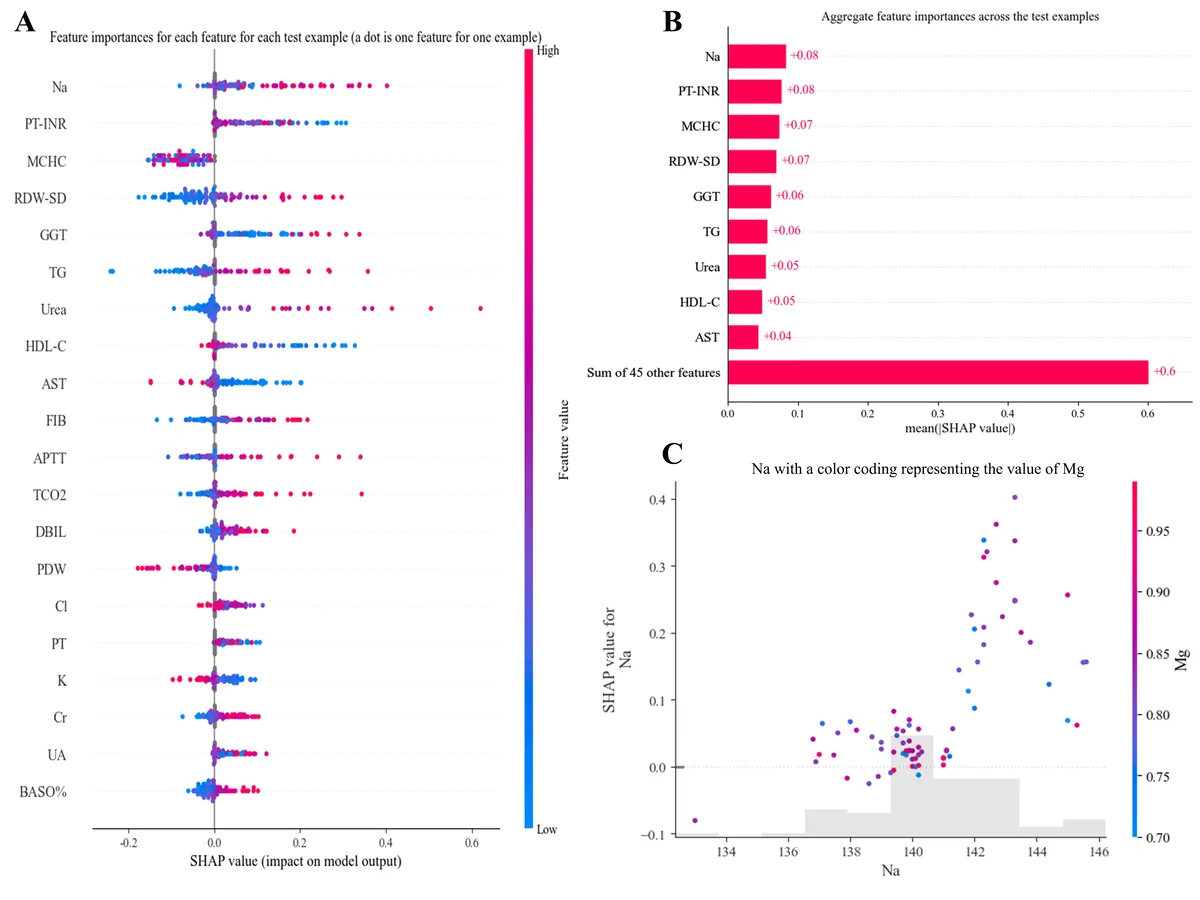
Model interpretability and feature importance analysis of the TabPFN model. (A) SHAP summary dot plot illustrating the impact of feature values on model output. Each dot represents a single prediction; red points indicate high feature values, and blue points indicate low feature values. (B) Bar plot of the mean absolute SHAP values ranking the global feature importance. (C) SHAP dependence plot for Sodium (Na), visualizing the non-linear relationship between sodium levels (x-axis) and predicted risk (SHAP value, y-axis), colored by the interaction feature Magnesium (Mg). APTT: activated partial thromboplastin time; AST: aspartate aminotransferase; BASO%: basophil percentage; Cl: chloride; Cr: creatinine; DBIL: direct bilirubin; FIB: fibrinogen; GGT: gamma-glutamyl transferase; HDL-C: high-density lipoprotein cholesterol; K: potassium; MCHC: mean corpuscular hemoglobin concentration; Mg: magnesium; Na: sodium; PDW: platelet distribution width; PT: prothrombin time; PT-INR: prothrombin time international normalized ratio; RDW-SD: red blood cell distribution width standard deviation; SHAP: Shapley Additive Explanations; TabPFN: Tabular Prior-Data Fitted Network; TCO2: total carbon dioxide; TG: triglycerides; UA: uric acid.

### Computational Efficiency

To evaluate the computational overhead, we measured the inference time of the proposed model. In our empirical testing on a standard desktop central processing unit (CPU), generating a risk prediction for a single patient—including data loading and processing—required approximately 1 second (~998 milliseconds).

## Discussion

### Principal Findings

This study developed and validated an interpretable TabPFN‑based screening model for CVA using routine laboratory data. The model demonstrated good discriminative performance across internal, temporal, and external validation sets, although some variation was observed across validation settings. Unlike the traditional diagnostic pathway requiring complex PFTs, this model relies solely on readily available hematological, biochemical, and coagulation markers. This offers a promising approach to address underdiagnosis in primary care.

### Comparison With Previous Work

Tree-based algorithms, such as XGBoost, remain strong benchmarks for tabular prediction tasks and frequently outperform deep learning approaches on conventional tabular datasets [18]. More recent tabular deep learning architectures have shown promising results, although their performance can vary in relatively small clinical datasets [19-21]. In this study, TabPFN and XGBoost exhibited different performance profiles across validation settings. TabPFN achieved higher AUCs in the internal testing and temporal validation cohorts than XGBoost (0.964 vs 0.890 and 0.925 vs 0.846, respectively), whereas XGBoost achieved a numerically higher AUC in the external validation cohort (0.912 vs 0.858). At the fixed probability threshold of 0.5, however, TabPFN showed higher external accuracy and specificity, whereas XGBoost showed higher sensitivity. These findings emphasize the distinction between threshold-independent discrimination and threshold-dependent classification performance. An additional methodological consideration is that post hoc isotonic calibration was applied to TabPFN but not to XGBoost. Consequently, direct comparison of accuracy, sensitivity, and specificity at a fixed probability threshold is methodologically asymmetric. In particular, the lower external specificity of XGBoost may partly reflect differences in probability calibration and threshold positioning rather than deterioration in ranking ability, which is consistent with its external AUC of 0.912. Accordingly, our findings should not be interpreted as demonstrating uniformly superior generalization of TabPFN over XGBoost. Instead, TabPFN showed more balanced threshold-dependent classification performance at the selected cutoff across the prospective validation cohorts, while XGBoost retained strong discriminative ability in the external cohort. Nevertheless, TabPFN remains methodologically attractive for small-to-medium-sized clinical tabular datasets. Foundation models represent an emerging direction in medical AI [22], and TabPFN specifically combines a pretrained transformer-based architecture with in-context learning, enabling predictions without conventional dataset-specific iterative model training or extensive hyperparameter optimization [15]. These characteristics may offer practical advantages in clinical settings with limited sample sizes. At the same time, TabPFN also showed a decrease in discrimination from the training cohort to the external validation cohort, with its AUC declining from 0.995 to 0.858. This finding underscores the need for further evaluation of both TabPFN and conventional tree-based models in larger and more geographically diverse cohorts.

Interpretability analysis provided critical insights into the biological underpinnings of the model’s decisions, highlighting serum sodium (Na) and PT-INR as the leading predictors, with MCHC and RDW-SD showing the same rounded mean absolute SHAP value at the reported 2-decimal precision. Specifically, the model revealed a non-linear trend where serum Na levels exceeding approximately 141 mmol/L were associated with higher predicted CVA risk. However, with a mean absolute SHAP value of 0.08, its absolute marginal effect remains modest. The wide variance around this level, which depends on concurrent magnesium concentrations, demonstrates a complex multielectrolyte interaction rather than a strict standalone clinical threshold. Nonetheless, this overall pattern aligns with the systemic pro-inflammatory effects of high dietary sodium load. Robust epidemiological evidence from the UK Biobank (n=42,976) has confirmed a positive association between sodium excretion and asthma incidence, noting that excess sodium aggravates both allergic Th2 and neutrophilic Th17 airway inflammation [23]. Beyond this association, mechanistic studies have demonstrated that elevated sodium induces SGK1 to stabilize the IL-23 receptor, promoting the differentiation of pathogenic Th17 cells, thereby providing a plausible mechanism for the persistent and steroid-resistant inflammation of CVA [24]. This multidimensional electrolyte interaction underscores that the model relies on holistic metabolic signatures rather than a strict, standalone clinical threshold. Concurrently, lower PT-INR emerged as a pivotal risk factor. Clinically, PT-INR assesses the extrinsic coagulation pathway. Lower values indicate a shortened clotting time and a hypercoagulable state. Previous studies have substantiated this link, demonstrating that tissue factor, the initiator of this pathway, increases during asthmatic inflammation to drive systemic coagulation [25]. Furthermore, the identification of erythrocyte indices, specifically RDW-SD and MCHC, supports the hypothesis that CVA involves broader systemic physiological dysregulation. As a classic marker of chronic inflammation and oxidative stress [26], red cell distribution width–derived markers have been identified as independent risk factors for asthma, with a mere 0.5-unit increase nearly doubling the disease risk (odds ratio 1.82, 95% CI 1.55-2.12) [27].

The clinical value of our model is highlighted when compared against traditional diagnostic methods reliant on single-indicator thresholds. Although previous studies have suggested the utility of individual biomarkers, such as FeNO or eosinophils, these often lack sufficient diagnostic sensitivity to serve as standalone screening tools. For instance, a systematic review and meta-analysis of 2187 patients revealed that while FeNO measurement offers moderate diagnostic accuracy, its pooled sensitivity remains low at 0.72, implying that nearly 30% of CVA cases could be missed if used as a sole rule-out test [28]. This limitation complicates clinical decision-making, particularly given that current European Respiratory Society (ERS) guidelines emphasize the need for accurate identification of CVA to guide targeted anti-inflammatory therapy [29]. By integrating nonlinear interactions among multiple metabolic and hematological features, our TabPFN model overcomes these single-marker limitations. Moreover, by identifying systemic predictors, such as Sodium and PT-INR, our study contributes new pathophysiological insights, highlighting that CVA screening can significantly benefit from assessing broader systemic metabolic and hypercoagulable states rather than relying solely on localized airway markers. Furthermore, DCA confirmed the clinical utility of the model, demonstrating higher net benefit than default strategies across broad threshold probability ranges in the validation cohorts. The observed ranges differed slightly between cohorts, consistent with differences in validation settings, following established methodology [30]. Practically, our model ensures minimal computational cost and robust out-of-the-box performance without requiring specialized hardware (eg, graphics processing units [GPUs]) or exhaustive hyperparameter tuning. It can be easily integrated into existing electronic health record (EHR) systems, making it highly feasible for rapid deployment in resource-limited primary care settings.

### Limitations

Several limitations warrant mention. First, the retrospective training data and the lack of universally available confirmatory tests for CVA may introduce selection bias and label noise. We mitigated these by strict adherence to ERS guidelines and temporal validation. However, future multicenter prospective cohorts are needed. Second, our temporal and external validation cohorts were relatively small due to the strict pulmonary testing criteria. The limited cohort sizes may reduce the precision of performance estimates and constrain generalizability, as also reflected by the corresponding confidence intervals. Larger multicenter prospective studies are needed to further validate these findings. Third, the full 54-variable model includes coagulation profiles that are not routinely tested in primary care. To address this, our feature pruning analysis (Table S5 in Multimedia Appendix 1) confirmed that a strictly routine 49-variable model still achieves high sensitivity (88.6%). This enables a practical 2-tier approach—using the 49-variable model for initial screening in primary care and reserving the full 54-variable model for further specialist evaluation in specialized centers [31]. Nevertheless, the pruned 49-variable model showed a marked drop in specificity to 55.8% in external validation, implying that approximately half of patients with non-CVA could be unnecessarily referred for further confirmatory testing. This high false-positive rate may compromise the cost-effectiveness of this primary-care screening strategy and warrants careful consideration in future implementation studies. Fourth, variables such as APTT had moderate missing rates. In real‑world practice, this missingness is often informative (missing not at random) because doctors order specific coagulation tests based on suspected disease severity. TabPFN natively processes raw missing values to capture this clinical intuition. However, traditional benchmarks like XGBoost were evaluated on KNN‑imputed data. This was due to upstream pipeline locks and the strict complete‑case requirements of downstream validation frameworks. Although comparing TabPFN against an imputed baseline introduces a methodological discrepancy, it accurately reflects the real‑world deployment constraints of traditional clinical algorithms. Finally, missing outpatient medication records introduce a potential confounding risk from drugs like proton pump inhibitors, diuretics, or anticoagulants. This bias was largely minimized by requiring complete PFT, which naturally excluded classic gastroesophageal reflux cough patients who are typically treated empirically without undergoing such evaluations. Additionally, TabPFN relies on high-dimensional interactions across 54 variables rather than isolated single-marker thresholds, providing resilience against localized drug-induced fluctuations. Future prospective studies with tracked medication registries remain necessary to completely decouple true biological signatures from treatment effects.

### Future Directions

Two main areas require further research. First, experimental studies are needed to establish the precise biological mechanisms linking electrolyte imbalances and coagulation dysfunction to CVA. Second, prospective implementation studies in primary care settings are recommended to integrate this model into EHRs and evaluate its real-world impact on diagnostic efficiency.

### Conclusions

This study prospectively validated an interpretable TabPFN model for CVA screening using routine laboratory data. TabPFN demonstrated robust performance across prospective validation cohorts and showed favorable threshold-dependent classification characteristics at the fixed cutoff. However, model comparisons were metric dependent; TabPFN achieved higher AUCs than XGBoost in the internal testing and temporal validation cohorts, whereas XGBoost achieved a higher AUC in the external validation cohort. These findings support TabPFN as a promising and accessible screening approach for CVA while avoiding claims of uniform superiority over conventional machine-learning models.

## Data Availability

The datasets generated and analyzed during this study are not publicly available due to ethical restrictions and patient privacy concerns (the data contain potentially identifying information). However, the de-identified participant dataset, study protocol, and statistical analysis code are available from the corresponding author upon reasonable request, subject to approval from the institutional ethics committee. The Python implementation code for the TabPFN and benchmark models is also available from the corresponding author upon reasonable request.

## Appendix

Multimedia Appendix 1 Additional data on proportions of missing data for each variable and sensitivity analysis.

Multimedia Appendix 2 Complete hyperparameter search grid and final selected parameter values.

## Abbreviations

APTT: activated partial thromboplastin time
AUC: area under the curve
AUROC: area under the receiver operating characteristic curve
CPU: central processing unit
CVA: cough variant asthma
DCA: decision curve analysis
EHR: electronic health record
ERS: European Respiratory Society
GPU: graphics processing unit
Hb: hemoglobin
KNN: k-nearest neighbors
ML: machine learning
PFT: pulmonary function test
PT-INR: prothrombin time international normalized ratio
RDW-SD: red cell distribution width-standard deviation
SHAP: Shapley Additive Explanations
TabPFN: Tabular Prior-Data Fitted Network
XGBoost: extreme gradient boosting
