# Supplementary material for: Screening for Cough Variant Asthma Using the TabPFN Model on Routine Laboratory Data in a Multicenter Cohort: Prospective Validation Study
Source: JMIR Med Inform. 2026 Oct 23;14:e92211. doi: 10.2196/92211 (PMC13743963; doi:10.2196/92211)
Supplement: Supplementary file 1 [file medinform_v14i1e92211_app1.docx]

**Multimedia Appendices**

Table S1. Clinical laboratory variables with the percentage of missing data. Pages 2-3

Table S2. Sensitivity analysis of KNN imputation methods with different hyperparameter K value. Page 4

Table S3. Comparison of demographics and laboratory test data among the validation cohorts. Pages 5-10

Table S4. Predictive performance of the TabPFN model across age subgroups. Page 11

Table S5. Predictive performance of the TabPFN model (coagulation indicators excluded). Page 12

Figure S1. Detailed performance evaluation of the TabPFN foundation model across all study cohorts. Page 13

Figure S2. Calibration curves of the TabPFN and XGBoost models across validation cohorts. Page 14

**Table S1: Clinical laboratory variables with the percentage of missing data.**

|  | **Variables** | **Total retrospective cohort** | | **Missing data** | | **Temporal validation (Internal)** | | **Missing data** | | **External validation** | **Missing data** | |
| --- | --- | --- | --- | --- | --- | --- | --- | --- | --- | --- | --- | --- |
|  |  |  |  | **Count** | **%** |  |  | **Count** | **%** |  | **Count** | **%** |
|  | **Biochemical**  **indicators** | | | | | |  |  | |  |  |  |
| A/G (ratio)  ALB (g/L)  ALP (U/L)  ALT (U/L)  AST (U/L)  Ca (mmol/L)  Cl (mmol/L)  Cr (μmol/L)  DBIL (μmol/L)  eGFR(ml/min/1.73m²)  GGT (U/L)  GLB (g/L)  Glu (mmol/L)  HDL-C (mmol/L)  K (mmol/L)  LDH (U/L)  LDL-Ch (mmol/L)  Mg (mmol/L)  Na (mmol/L)  TBIL (μmol/L)  TCO2 (mmol/L)  TG (mmol/L)  TP (g/L)  UA (μmol/L)  Urea (mmol/L) | | | 483  497  484  496  479  474  469  495  502  489  448  480  472  368  479  436  453  411  465  502  446  441  482  492  487 | 50  36  49  37  54  59  64  38  31  44  85  53  61  165  54  97  80  122  68  31  87  92  51  41  46 | 9.38%  6.75%  9.19%  6.94%  10.13%  11.07%  12.01%  7.13%  5.82%  8.26%  15.95%  9.94%  11.44%  30.96%  10.13%  18.20%  15.01%  22.89%  12.76%  5.82%  16.32%  17.26%  9.57%  7.69%  8.63% | | 55  55  54  55  52  53  53  51  56  46  51  54  47  38  53  46  46  41  50  56  44  46  54  51  52 | 4  4  5  4  7  6  6  8  3  13  8  5  12  21  6  13  13  18  9  3  15  13  5  8  7 | 6.78%  6.78%  8.47%  6.78%  11.86%  10.17%  10.17%  13.56%  5.08%  22.03%  13.56%  8.47%  20.34%  35.59%  10.17%  22.03%  22.03%  30.51%  15.25%  5.08%  25.42%  22.03%  8.47%  13.56%  11.86% | 71  71  77  71  71  68  68  74  77  66  68  71  71  71  66  58  64  69  67  76  70  72  67  70  70 | 7  7  1  7  7  10  10  4  1  12  10  7  7  7  12  20  14  9  11  2  8  6  11  8  8 | 8.97%  8.97%  1.28%  8.97%  8.97%  12.82%  12.82%  5.13%  1.28%  15.38%  12.82%  8.97%  8.97%  8.97%  15.38%  25.64%  17.95%  11.54%  14.10%  2.56%  10.26%  7.69%  14.10%  10.26%  10.26% |
| **Coagulation indicators** | | |  |  |  | |  |  |  |  |  |  |
| APTT (s)  D-Dimer (mg/L)  FIB (g/L)  PT (s)  PT-INR (ratio) | | | 336  452  368  448  453 | 197  81  165  85  80 | 36.96%  15.20%  30.96%  15.95%  15.01% | | 37  45  48  50  48 | 22  14  11  9  11 | 37.29%  23.73%  18.64%  15.25%  18.64% | 74  77  72  73  73 | 4  1  6  5  5 | 5.13%  1.28%  7.69%  6.41%  6.41% |
| **Complete blood count indicators** | | |  |  |  | |  |  |  |  |  |  |
| BASO# (×10^9/L)  BASO% (%)  EOS# (×10^9/L)  EOS% (%)  Hb (g/L)  HCT (%)  LYM# (×10^9/L)  LYM% (%)  MCH (pg)  MCHC (g/L)  MCV (fL)  Mono# (×10^9/L)  Mono% (%)  MPV (fL)  Neu# (×10^9/L)  Neu% (%)  PCT (%)  PDW (%)  P-LCR (%)  PLT (×10^9/L)  RBC (×10^12/L)  RDW-CV (%)  RDW-SD (fL)  WBC (×10^9/L) | | | 519  519  511  511  529  530  509  519  520  520  520  519  519  514  530  530  520  516  518  525  521  528  514  531 | 14  14  22  22  4  3  24  14  13  13  13  14  14  19  3  3  13  17  15  8  12  5  19  2 | 2.63%  2.63%  4.13%  4.13%  0.75%  0.56%  4.50%  2.63%  2.44%  2.44%  2.44%  2.63%  2.63%  3.56%  0.56%  0.56%  2.44%  3.19%  2.81%  1.50%  2.25%  0.94%  3.56%  0.38% | | 56  56  54  54  56  55  55  56  55  56  56  56  56  56  56  56  56  56  56  56  56  56  56  57 | 3  3  5  5  3  4  4  3  4  3  3  3  3  3  3  3  3  3  3  3  3  3  3  2 | 5.08%  5.08%  8.47%  8.47%  5.08%  6.78%  6.78%  5.08%  6.78%  5.08%  5.08%  5.08%  5.08%  5.08%  5.08%  5.08%  5.08%  5.08%  5.08%  5.08%  5.08%  5.08%  5.08%  3.39% | 75  74  74  76  78  74  77  76  75  76  76  75  75  75  75  75  69  69  69  75  75  75  60  75 | 3  4  4  2  0  4  1  2  3  2  2  3  3  3  3  3  9  9  9  3  3  3  18  3 | 3.85%  5.13%  5.13%  2.56%  0.00%  5.13%  1.28%  2.56%  3.85%  2.56%  2.56%  3.85%  3.85%  3.85%  3.85%  3.85%  11.54%  11.54%  11.54%  3.85%  3.85%  3.85%  23.08%  3.85% |

A/G: albumin-to-globulin ratio; ALB: albumin; ALP: alkaline phosphatase; ALT: alanine aminotransferase; APTT: activated partial thromboplastin time; AST: aspartate aminotransferase; BASO: basophil; Ca: calcium; Cl: chloride; Cr: creatinine; DBIL: direct bilirubin; D-Dimer: D-dimer; eGFR: estimated glomerular filtration rate; EOS: eosinophil; FIB: fibrinogen; GGT: gamma-glutamyl transferase; GLB: globulin; Glu: glucose; Hb: hemoglobin; HCT: hematocrit; HDL-C: high-density lipoprotein cholesterol; K: potassium; LDH: lactate dehydrogenase; LDL-Ch: low-density lipoprotein cholesterol; LYM: lymphocyte; MCH: mean corpuscular hemoglobin; MCHC: mean corpuscular hemoglobin concentration; MCV: mean corpuscular volume; Mg: magnesium; Mono: monocyte; MPV: mean platelet volume; Na: sodium; Neu: neutrophil; PCT: plateletcrit; PDW: platelet distribution width; P-LCR: platelet-large cell ratio; PLT: platelets; PT: prothrombin time; PT-INR: prothrombin time international normalized ratio; RBC: red blood cell; RDW-CV: red cell distribution width-coefficient of variation; RDW-SD: red cell distribution width-standard deviation; TBIL: total bilirubin; TCO2: total carbon dioxide; TG: triglyceride; TP: total protein; UA: uric acid; Urea: blood urea nitrogen; WBC: white blood cell.

**Table S2: Sensitivity analysis of KNN imputation methods with different hyperparameter K value.**

| **Models** | **Overall**  **Accuracy** | **ROC AUC**  **(95% CI)** | **Sensitivity** | **Specificity** | **Precision** | **F1** |
| --- | --- | --- | --- | --- | --- | --- |
| K=5 | 0.790 | 0.891 (0.826–0.944) | 0.782 | 0.8 | 0.811 | 0.796 |
| K=10 | 0.762 | 0.888 (0.822–0.941) | 0.691 | 0.84 | 0.826 | 0.752 |
| K=15 | 0.829 | 0.890 (0.820–0.947) | 0.8 | 0.86 | 0.863 | 0.83 |

KNN: K-nearest neighbors; ROC: receiver operating characteristic; AUC: area under the curve; CI: confidence interval.

**Table S3: Comparison of demographics and laboratory test data among the validation cohorts.**

**Table S3a. Baseline characteristics of study cohorts**

| **Variables** | | **Total retrospective cohort (n=533)** | **Temporal validation**  **(n=59)** | **External validation (n=78)** |
| --- | --- | --- | --- | --- |
| **Outcome, n (%)** | |  |  |  |
| CVA (Case) | | 258 (48.40%) | 27 (45.76%) | 35 (44.87%) |
| Non-CVA (Control) | | 275 (51.60%) | 32 (54.24%) | 43 (55.13%) |
| Sex, n (%) | |  |  |  |
| Male | | 244 (45.78%) | 28(47.46%) | 37(47.43%) |
| Female | | 289 (54.22%) | 31(52.54%) | 41(52.56%) |
| Age (years) | | 50.00 [38.00-62.00] | 48.00 [36.50 - 57.50] | 44.50 [34.25 - 55.00] |
| **Biochemical indicators** | | | | |
| A/G (ratio) | 1.59 [1.45-1.77] | | 1.63 [1.49 - 1.80] | 1.57 [1.39 - 1.69] |
| ALB (g/L) | 41.60 [38.80-44.50] | | 42.40 [39.70 - 45.50] | 42.60 [38.90 - 45.60] |
| ALP (U/L) | 69.00 [54.00-86.00] | | 72.00 [56.73 - 85.75] | 65.50 [51.00 - 80.00] |
| ALT (U/L) | 17.00 [12.00-23.00] | | 15.80 [12.50 - 21.60] | 16.00 [11.00 - 24.00] |
| AST (U/L) | 20.00 [17.00-26.45] | | 18.80 [16.00 - 20.63] | 18.20 [16.00 - 23.00] |
| Ca (mmol/L) | 2.23 [2.15-2.32] | | 2.27 [2.20 - 2.38] | 2.27 [2.15 - 2.32] |
| Cl (mmol/L) | 107.00 [105.30-108.60] | | 107.60 [106.70 - 108.80] | 104.65 [103.00 - 107.20] |
| Cr (μmol/L) | 59.00 [51.00-70.00] | | 58.00 [53.50 - 71.50] | 66.47 [57.43 - 79.75] |
| DBIL (μmol/L) | 2.60 [1.80-3.60] | | 2.60 [2.08 - 3.65] | 2.50 [1.40 - 3.80] |
| eGFR(ml/min/1.73m²) | 100.62 [90.96-112.15] | | 99.36 [89.25 - 115.01] | 115.56 [99.47 - 122.59] |
| GGT (U/L) | 20.00 [14.00-28.00] | | 16.00 [11.93 - 28.00] | 18.00 [13.25 - 27.00] |
| GLB (g/L) | 26.00 [23.80-28.90] | | 25.80 [23.93 - 28.00] | 27.90 [24.95 - 30.25] |
| Glu (mmol/L) | 4.89 [4.48-5.45] | | 4.81 [4.51 - 5.32] | 4.82 [4.51 - 5.54] |
| HDL-C (mmol/L) | 1.17 [0.99-1.43] | | 1.19 [0.91 - 1.43] | 1.13 [1.02 - 1.41] |
| K (mmol/L) | 3.95 [3.74-4.21] | | 4.00 [3.80 - 4.26] | 3.88 [3.70 - 4.05] |
| LDH (U/L) | 196.00 [170.00-223.25] | | 193.00 [169.25 - 230.00] | 194.75 [162.10 - 230.25] |
| LDL-Ch (mmol/L) | 2.50 [2.03-2.99] | | 2.19 [2.01 - 2.86] | 2.27 [2.07 - 2.73] |
| Mg (mmol/L) | 0.84 [0.80-0.89] | | 0.82 [0.78 - 0.89] | 0.85 [0.82 - 0.94] |
| Na (mmol/L) | 140.80 [139.60-142.10] | | 141.25 [139.73 - 142.93] | 141.00 [139.00 - 142.15] |
| TBIL (μmol/L) | 10.50 [6.63-13.50] | | 9.70 [7.00 - 12.93] | 9.95 [7.38 - 13.93] |
| TCO2 (mmol/L) | 24.90 [23.10-26.48] | | 24.45 [23.60 - 25.38] | 22.90 [20.73 - 26.03] |
| TG (mmol/L) | 1.23 [0.84-1.68] | | 0.96 [0.51 - 1.29] | 0.74 [0.57 - 1.16] |
| TP (g/L) | 68.20 [64.00-71.34] | | 68.60 [64.50 - 71.95] | 70.50 [65.30 - 74.10] |
| UA (μmol/L) | 305.00 [258.00-357.00] | | 294.00 [236.50 - 351.50] | 286.00 [229.78 - 336.15] |
| Urea (mmol/L) | 5.16 [4.29-6.10] | | 4.86 [3.92 - 5.81] | 4.52 [4.05 - 5.77] |
| **Coagulation indicators** | | | | |
| APTT (s) | | 27.70 [25.90-30.10] | 28.20 [25.60 - 30.00] | 30.45 [28.30 - 32.69] |
| D-Dimer (mg/L) | | 0.27 [0.18-0.45] | 0.34 [0.26 - 0.52] | 0.26 [0.17 - 0.40] |
| FIB (g/L) | | 2.86 [2.45-3.53] | 3.09 [2.50 - 3.80] | 3.24 [2.63 - 3.69] |
| PT (s) | | 11.40 [10.90-12.00] | 11.40 [10.75 - 12.40] | 11.70 [11.20 - 12.00] |
| PT-INR (ratio) | | 0.98 [0.93-1.04] | 0.97 [0.91 - 1.07] | 1.07 [1.02 - 1.11] |
| **Complete blood count indicators** | | | | |
| BASO# (×10^9/L) | | 0.03 [0.02-0.04] | 0.03 [0.02 - 0.04] | 0.03 [0.01 - 0.05] |
| BASO% (%) | | 0.50 [0.30-0.70] | 0.50 [0.30 - 0.61] | 0.51 [0.30 - 0.78] |
| EOS# (×10^9/L) | | 0.13 [0.08-0.23] | 0.13 [0.08 - 0.25] | 0.11 [0.07 - 0.30] |
| EOS% (%) | | 2.30 [1.50-3.70] | 2.45 [1.23 - 4.20] | 2.01 [1.18 - 4.40] |
| Hb (g/L) | | 132.00 [124.00-140.00] | 132.50 [123.50 - 145.00] | 127.00 [120.05 - 138.75] |
| HCT (%) | | 44.00 [41.00–47.00] | 40.00 [38.00–45.00] | 39.00 [36.00–43.00] |
| LYM# (×10^9/L) | | 1.86 [1.49-2.36] | 1.83 [1.38 - 2.19] | 1.77 [1.41 - 2.20] |
| LYM% (%) | | 30.70 [25.30-36.25] | 29.10 [23.90 - 36.18] | 31.55 [24.58 - 35.40] |
| MCH (pg) | | 30.10 [28.88-31.13] | 29.70 [28.45 - 30.90] | 31.00 [29.50 - 31.70] |
| MCHC (g/L) | | 330.00 [320.00-340.00] | 330.00 [320.00-340.00] | 338.45 [331.77-343.05] |
| MCV (fL) | | 90.30 [87.40-93.30] | 89.65 [86.95 - 92.35] | 91.15 [88.70 - 94.25] |
| Mono# (×10^9/L) | | 0.48 [0.37-0.61] | 0.46 [0.37 - 0.56] | 0.48 [0.39 - 0.58] |
| Mono% (%) | | 7.80 [6.30-9.30] | 7.05 [6.28 - 8.54] | 8.20 [6.60 - 9.35] |
| MPV (fL) | | 10.30 [9.80-10.90] | 10.50 [9.90 - 10.90] | 8.70 [8.05 - 9.72] |
| Neu# (×10^9/L) | | 3.57 [2.76-4.50] | 3.61 [2.86 - 4.72] | 3.47 [2.52 - 4.51] |
| Neu% (%) | | 57.07 [50.00-63.41] | 58.55 [51.88 - 65.13] | 59.40 [52.50 - 63.95] |
| PCT (%) | | 0.20 [0.20-0.30] | 0.30 [0.20 - 0.30] | 0.20 [0.20 - 0.30] |
| PDW (%) | | 12.10 [10.80-13.80] | 12.00 [11.08 - 12.95] | 12.00 [11.00 - 13.70] |
| P-LCR (%) | | 28.30 [23.73-33.40] | 28.25 [24.18 - 31.55] | 28.60 [23.90 - 33.40] |
| PLT (×10^9/L) | | 233.00 [192.00-288.00] | 231.50 [202.00 - 276.25] | 234.00 [187.90 - 268.00] |
| RBC (×10^12/L) | | 4.39 [4.13-4.72] | 4.60 [4.19 - 4.84] | 4.18 [3.82 - 4.52] |
| RDW-CV (%) | | 13.00 [12.00–13.00] | 13.00 [12.00–13.00] | 13.00 [13.00–14.00] |
| RDW-SD (fL) | | 41.90 [40.20-45.20] | 41.00 [39.48 - 43.33] | 41.30 [40.28 - 43.30] |
| WBC (×10^9/L) | | 6.28 [5.22-7.73] | 6.38 [5.07 - 7.52] | 5.91 [5.03 - 6.89] |

**Table S3b. Baseline characteristics stratified by outcome (CVA vs Non-CVA)**

| **Variables** | | **Total retrospective cohort (n=533)** | | | | | **Temporal validation**  **(n=59)** | | **External validation (n=78)** | | | | | | | | | |
| --- | --- | --- | --- | --- | --- | --- | --- | --- | --- | --- | --- | --- | --- | --- | --- | --- | --- | --- |
|  | | CVA | | | Non-CVA | | CVA | Non-CVA | CVA | Non-CVA | | | | | | | | |
| **Sex, n (%)** | |  | | | | |  | |  | | | | | | | | | |
| Male | | 114 (44.19%) | | | 130 (47.27%) | | 15(55.56%) | 13(40.62%) | 15(42.86%) | | | | | | 22(51.16%) | | | |
| Female | | 144 (55.81%) | | | 145 (52.73%) | | 12(44.44%) | 19(59.38%) | 20(57.14%) | | | | | | 21(48.84%) | | | |
| **Age (years)** | | 43.50 [33.00-55.00] | | | 57.00 [46.00-65.00] | | 48.00 [37.00-58.00] | 47.50 [36.25-56.25] | 48.00 [40.50-61.00] | | | | | | 40.00 [31.50-52.00] | | | |
| **Biochemical indicators** | | | | | | | | | | | | | | | | | | |
| A/G (ratio) | 1.57 [1.41-1.78] | | 1.60 [1.46-1.77] | | | | 1.66 [1.46-1.82] | 1.59 [1.50-1.78] | 1.51 [1.39-1.69] | | | 1.60 [1.40-1.70] | | | | | |  |
| ALB (g/L) | 42.45 [39.30-45.23] | | | 41.20 [38.40-43.60] | | | 45.15 [41.33-47.50] | 41.30 [38.80-42.80] | 40.00 [37.60-45.60] | | | | | 42.95 [39.85-45.58] | | | |  |
| ALP (U/L) | 67.00 [52.00-88.00] | | | 71.00 [59.00-84.50] | | | 75.00 [65.90-89.25] | 66.00 [51.75-82.75] | 65.25 [52.40-87.75] | | | | | 67.00 [46.00-74.50] | | | |  |
| ALT (U/L) | 17.00 [13.00-23.75] | | | 16.00 [12.00-22.75] | | | 15.15 [13.00-16.00] | 16.00 [12.00-26.00] | 15.00 [9.40-24.00] | | | | | 16.50 [13.25-23.00] | | | |  |
| AST (U/L) | 22.00 [17.50-30.13] | | | 19.00 [16.00-24.00] | | | 18.30 [15.95-20.00] | 19.00 [16.75-22.00] | 19.00 [16.00-24.10] | | | | | 18.15 [16.00-23.00] | | | |  |
| Ca (mmol/L) | 2.25 [2.15-2.35] | | | 2.22 [2.15-2.30] | | | 2.32 [2.22-2.45] | 2.23 [2.16-2.34] | 2.19  [2.14-2.31] | | | | | 2.30 [2.22-2.34] | | | |  |
| Cl (mmol/L) | 106.70 [104.93-109.10] | | | 107.20 [105.75-108.50] | | | 107.70 [106.65-109.55] | 107.40 [106.90-108.50] | 106.90 [103.00-108.00] | | | | | 104.50 [102.95-105.90] | | | |  |
| Cr (μmol/L) | 56.00 [50.00-65.75] | | 63.00 [54.00-73.00] | | | 57.00 [51.10-70.50] | | 60.00 [54.75-71.00] | 63.00 [53.45-80.15] | | | | | 71.60 [60.20-78.50] | | | |  |
| DBIL (μmol/L) | 2.65 [1.90-3.60] | | 2.60 [1.60-3.60] | | | 2.41 [2.13-3.43] | | 2.75 [2.08-3.88] | 2.58 [2.00-3.48] | | | | 2.12 [1.20-4.11] | | | | |  |
| eGFR(ml/min/1.73m²) | 106.32 [93.16-115.71] | | 97.45 [88.50-106.05] | | | 102.83 [87.69-117.05] | | 98.06 [90.47-108.74] | 118.26 [102.23-122.40] | | | | 115.46 [96.93-122.65] | | | | |  |
| GGT (U/L) | 20.50 [14.00-28.00] | | 20.00 [14.00-29.00] | | | 15.80 [11.76-28.00] | | 16.00 [12.50-28.50] | 18.75 [13.25-24.90] | | 18.00 [13.25-28.25] | | | | | | |  |
| GLB (g/L) | 26.40 [24.10-29.90] | | 25.40 [23.60-27.88] | | | 26.00 [24.13-29.88] | | 25.30 [23.85-27.85] | 27.90 [24.40-30.90] | | | | 27.80 [25.40-30.08] | | | |  |  |
| Glu (mmol/L) | 4.93 [4.51-5.52] | | 4.82 [4.44-5.41] | | | 4.85 [4.75-5.37] | | 4.59 [4.39-5.24] | 5.15 [4.69-5.78] | | | | 4.70 [4.41-5.36] | | | |  |  |
| HDL-C (mmol/L) | 1.21 [1.02-1.45] | | 1.11 [0.96-1.32] | | | 1.44 [0.88-1.67] | | 1.04 [0.95-1.32] | 1.25 [1.08-1.53] | | | | 1.06 [0.97-1.22] | | | |  |  |
| K (mmol/L) | 4.05 [3.81-4.36] | | 3.92 [3.70-4.10] | | | 4.19 [3.95-4.63] | | 3.92 [3.69-4.12] | 3.97 [3.77-4.29] | | | | 3.80 [3.68-3.93] | | | |  |  |
| LDH (U/L) | 195.00 [165.00-219.00] | | 198.00 [173.00-226.00] | | | 212.00 [185.00-279.00] | | 189.00 [168.50-204.50] | 199.00 [162.40-261.00] | | | | 180.00 [162.00-219.00] | | | |  |  |
| LDL-Ch (mmol/L) | 2.54 [2.10-2.95] | | 2.48 [1.98-3.03] | | | 2.28 [2.09-2.93] | | 2.06 [1.82-2.84] | 2.34 [2.10-2.69] | | | | 2.25 [1.94-2.73] | | | |  |  |
| Mg (mmol/L) | 0.84 [0.79-0.91] | | 0.84 [0.80-0.87] | | | 0.88 [0.75-0.92] | | 0.82 [0.78-0.85] | 0.84 [0.81-0.89] | | | | 0.90 [0.83-0.98] | | | |  |  |
| Na (mmol/L) | 140.20 [139.30-141.80] | | 141.15 [139.88-142.33] | | | 141.20 [139.10-143.00] | | 141.30 [139.90-142.70] | 140.40 [138.38-141.78] | | | | 141.00 [139.60-143.00] | | | |  |  |
| TBIL (μmol/L) | 11.85 [8.40-13.60] | | 8.95 [5.60-12.90] | | | 11.90 [7.41-13.88] | | 9.00 [7.03-12.40] | 11.35 [9.38-14.00] | | | 8.55 [6.93-13.23] | | | | |  |  |
| TCO2 (mmol/L) | 24.50 [22.80-25.58] | | 25.30 [23.48-27.03] | | | 24.00 [22.80-24.55] | | 24.95 [24.18-26.65] | 22.70 [20.45-25.23] | | | 22.90 [20.95-26.35] | | | | |  |  |
| TG (mmol/L) | 1.01 [0.75-1.40] | | 1.47 [1.02-2.06] | | | 0.54 [0.44-0.89] | | 1.10 [0.96-1.56] | 0.95 [0.65-1.17] | | | | 0.66 [0.53-1.14] | | | |  |  |
| TP (g/L) | 68.75 [65.63-72.20] | | 66.80 [62.70-71.10] | | | 70.20 [68.50-73.80] | | 66.70 [62.80-69.10] | 67.90 [65.90-74.10] | | | | 70.95 [65.15-74.08] | | | |  |  |
| UA (μmol/L) | 300.00 [255.50-320.00] | | 316.00 [263.00-380.00] | | | 296.50 [210.50-324.50] | | 289.00 [248.50-353.50] | 289.30 [232.10-314.50] | | | | 277.00 [229.00-348.90] | | | |  |  |
| Urea (mmol/L) | 5.07 [4.23-5.75] | | 5.37 [4.31-7.74] | | | 4.92 [3.87-5.46] | | 4.81 [4.03-6.06] | 4.64 [3.99-5.80] | | | | 4.50 [4.17-5.56] | | | |  |  |
| **Coagulation indicators** | | | | | | | | | | | | | | | | |  |  |
| APTT (s) | | 27.40  [25.30–28.50] | | | 29.50 [27.00-31.55] | 27.40 [25.80-29.10] | | 29.00 [25.48-31.18] | 28.80 [27.00-30.50] | | | | 31.40 [29.90-33.50] | | | |  |  |
| D-Dimer (mg/L) | | 0.26 [0.18-0.44] | | | 0.28 [0.19-0.45] | 0.35 [0.22-0.54] | | 0.34 [0.28-0.51] | 0.26 [0.16-0.36] | | | | 0.26 [0.19-0.47] | | | |  |  |
| FIB (g/L) | | 2.61 [2.34-3.05] | | | 3.18 [2.72-3.97] | 2.99 [2.11-3.64] | | 3.18 [2.71-3.83] | 2.77 [2.49-3.52] | | | 3.38 [2.93-3.88] | | | | |  |  |
| PT (s) | | 11.40 [10.80-11.80] | | | 11.50 [10.90-12.10] | 11.95 [11.08-13.05] | | 11.20 [10.70-12.10] | 11.55 [11.10-12.25] | | | 11.70 [11.40-12.00] | | | | |  |  |
| PT-INR (ratio) | | 0.97 [0.93-1.03] | | | 0.99 [0.93-1.05] | 1.05 [0.95-1.12] | | 0.94 [0.88-1.01] | 1.03 [1.00-1.12] | | | 1.08 [1.04-1.10] | | | | |  |  |
| **Complete blood count indicators** | | | | | | | | | | | | | | | | |  |  |
| BASO# (×10^9/L) | | 0.03 [0.02-0.04] | | | 0.03 [0.02-0.05] | 0.03 [0.02-0.05] | | 0.03 [0.02-0.04] | 0.03 [0.01-0.04] | | 0.03 [0.00-0.05] | | | | | |  |  |
| BASO% (%) | | 0.40 [0.30-0.60] | | | 0.50 [0.30-0.70] | 0.50 [0.33-0.60] | | 0.50 [0.30-0.70] | 0.51 [0.23-0.70] | | 0.56 [0.40-0.83] | | | | | |  |  |
| EOS# (×10^9/L) | | 0.13 [0.07-0.26] | | | 0.13 [0.08-0.22] | 0.14 [0.07-0.35] | | 0.13 [0.10-0.21] | 0.11 [0.07-0.27] | | 0.15 [0.08-0.30] | | | | | |  |  |
| EOS% (%) | | 2.30 [1.50-3.55] | | | 2.40 [1.60-3.73] | 2.90 [1.10-4.80] | | 2.40 [1.50-3.30] | 1.95 [1.06-4.13] | | 2.06 [1.30-4.65] | | | | | |  |  |
| Hb (g/L) | | 133.00 [126.00-141.00] | | | 132.00 [122.00-138.00] | 139.00 [125.50-150.25] | | 128.50 [122.00-139.75] | 128.20 [120.15-137.00] | | | 125.90 [120.10-139.50] | | | | |  |  |
| HCT (%) | | 45.00 [42.00–47.00] | | | 44.00 [41.00–46.00] | 43.00 [38.00–47.00] | | 40.00 [37.00–41.00] | 39.00 [37.00–43.00] | | | 38.00 [35.00–41.00] | | | | |  |  |
| LYM# (×10^9/L) | | 1.99 [1.61-2.55] | | | 1.77 [1.40-2.16] | 1.61 [1.30-2.29] | | 1.92 [1.52-2.19] | 1.79 [1.47-2.32] | | | 1.76 [1.41-2.16] | | | | |  |  |
| LYM% (%) | | 30.80 [25.30-35.33] | | | 30.60 [25.30-37.35] | 28.10 [18.35-31.13] | | 29.85 [27.68-37.45] | 30.95 [23.15-35.25] | | | 31.95 [27.93-35.30] | | | |  |  |  |
| MCH (pg) | | 30.00 [28.70-30.90] | | | 30.20 [28.90-31.40] | 29.40 [28.30-30.60] | | 29.70 [28.65-30.98] | 30.70 [29.40-31.20] | | | 31.30 [29.73-32.30] | | | |  |  |  |
| MCHC (g/L) | | 330.00 [320.00-340.00] | | | 330.00 [320.00-340.00] | 330.00 [320.00-340.00] | | 330.00 [320.00-330.00] | 338.15 [331.30-342.88] | | | 338.75 [332.33-344.00] | | | |  |  |  |
| MCV (fL) | | 89.70 [86.20-92.40] | | | 91.10 [88.45-94.40] | 89.35 [86.73-90.38] | | 90.30 [88.30-92.60] | 90.25 [88.25-92.08] | | | 92.15 [89.53-95.00] | | | |  |  |  |
| Mono# (×10^9/L) | | 0.51 [0.39-0.62] | | | 0.46 [0.36-0.60] | 0.56 [0.41-0.69] | | 0.43 [0.36-0.48] | 0.49 [0.34-0.59] | | | 0.47 [0.40-0.58] | | | |  |  |  |
| Mono% (%) | | 7.40 [6.00-9.00] | | | 8.00 [6.60-9.50] | 7.35 [6.23-9.31] | | 6.95 [6.33-8.48] | 7.54 [6.10-8.88] | | | 8.70 [7.20-9.40] | | | |  |  |  |
| MPV (fL) | | 10.20 [9.60-10.80] | | | 10.40 [9.90-11.10] | 10.50 [10.20-10.98] | | 10.35 [9.90-10.88] | 9.25 [8.63-10.05] | | | 8.30 [7.80-9.20] | | | |  |  |  |
| Neu# (×10^9/L) | | 3.74 [2.99-4.79] | | | 3.40 [2.65-4.27] | 4.27 [3.18-5.39] | | 3.23 [2.56-3.93] | 3.80 [2.98-4.94] | | | 3.10 [2.40-3.90] | | | |  |  |  |
| Neu% (%) | | 57.08 [49.36-64.11] | | | 57.06 [50.39-63.30] | 62.97 [53.80-70.46] | | 57.40 [51.27-61.89] | 60.95 [53.58-68.85] | | | 58.40 [52.00-62.20] | | | |  |  |  |
| PCT (%) | | 0.30 [0.20-0.30] | | | 0.20 [0.20 - 0.30] | 0.20 [0.20 - 0.30] | | 0.30 [0.20-0.30] | 0.20 [0.20-0.30] | | 0.30 [0.20-0.30] | | | | | |  |  |
| PDW (%) | | 12.20 [10.80-14.10] | | | 12.00 [10.80-13.25] | 12.00 [11.20-12.90] | | 11.80 [10.95-13.08] | 12.00 [11.10-14.30] | | 11.90 [11.03-12.85] | | | | | |  |  |
| P-LCR (%) | | 28.30 [23.40-33.40] | | | 28.20 [24.00-33.35] | 28.70 [26.43-32.10] | | 26.95 [23.73-31.43] | 28.80 [24.50-34.20] | | 28.15 [23.90-32.75] | | | | | |  |  |
| PLT (×10^9/L) | | 245.00 [204.00-296.00] | | | 223.50 [183.00-277.00] | 228.50 [205.75-280.00] | | 234.00 [197.50-270.00] | 233.00 [196.50-268.00] | | 235.00 [178.00-267.00] | | | | | |  |  |
| RBC (×10^12/L) | | 4.45 [4.19-4.81] | | | 4.35 [4.02-4.64] | 4.68 [4.40-4.97] | | 4.37 [4.12-4.71] | 4.19 [3.98-4.54] | | 4.18 [3.74-4.50] | | | | | |  |  |
| RDW-CV (%) | | 13.00 [12.00–13.00] | | | 13.00 [12.00–13.00] | 13.00 [12.00–13.00] | | 13.00 [12.00–13.00] | 13.00 [13.00–14.00] | | 13.00 [13.00–14.00] | | | | | |  |  |
| RDW-SD (fL) | | 41.25 [39.70-42.38] | | | 43.80 [40.88-49.18] | 40.30 [38.53-42.98] | | 41.25 [39.85-44.23] | 41.20 [40.43-41.88] | | 41.90 [40.28-44.03] | | | | | |  |  |
| WBC (×10^9/L) | | 6.60 [5.62-7.86] | | | 5.96 [4.90-7.58] | 6.74 [5.30-8.15] | | 6.00 [5.03-7.04] | 6.20 [5.39-6.98] | | 5.86 [4.51-6.80] | | | | | |  |  |

A/G: albumin-to-globulin ratio; ALB: albumin; ALP: alkaline phosphatase; ALT: alanine aminotransferase; APTT: activated partial thromboplastin time; AST: aspartate aminotransferase; BASO: basophil; Ca: calcium; Cl: chloride; Cr: creatinine; DBIL: direct bilirubin; D-Dimer: D-dimer; eGFR: estimated glomerular filtration rate; EOS: eosinophil; FIB: fibrinogen; GGT: gamma-glutamyl transferase; GLB: globulin; Glu: glucose; Hb: hemoglobin; HCT: hematocrit; HDL-C: high-density lipoprotein cholesterol; K: potassium; LDH: lactate dehydrogenase; LDL-Ch: low-density lipoprotein cholesterol; LYM: lymphocyte; MCH: mean corpuscular hemoglobin; MCHC: mean corpuscular hemoglobin concentration; MCV: mean corpuscular volume; Mg: magnesium; Mono: monocyte; MPV: mean platelet volume; Na: sodium; Neu: neutrophil; PCT: plateletcrit; PDW: platelet distribution width; P-LCR: platelet-large cell ratio; PLT: platelets; PT: prothrombin time; PT-INR: prothrombin time international normalized ratio; RBC: red blood cell; RDW-CV: red cell distribution width-coefficient of variation; RDW-SD: red cell distribution width-standard deviation; TBIL: total bilirubin; TCO2: total carbon dioxide; TG: triglyceride; TP: total protein; UA: uric acid; Urea: blood urea nitrogen; WBC: white blood cell.

**Table S4:** Predictive performance of the TabPFN model across age subgroups.

| **Dataset** | **N** | **CVA, n** | **Non-CVA, n** | **Overall Accuracy** | **ROC AUC  (95% CI)** | **Sensitivity** | **Specificity** | **Precision** | **F1  Score** |
| --- | --- | --- | --- | --- | --- | --- | --- | --- | --- |
| **Temporal Validation**  **(<50 years)** | 32 | 14 | 18 | 81.3% | 0.909 (0.797–0.991) | 85.7% | 77.8% | 75.0% | 0.800 |
| **Temporal Validation**  **(≥50 years)** | 27 | 13 | 14 | 85.2% | 0.945 (0.846–1.000) | 69.2% | 100.0% | 100.0% | 0.818 |
| **External Validation**  **(<50 years)** | 48 | 19 | 29 | 79.2% | 0.869 (0.739–0.971) | 78.9% | 79.3% | 71.4% | 0.750 |
| **External Validation**  **(≥50 years)** | 30 | 16 | 14 | 80.0% | 0.866 (0.713–0.982) | 62.5% | 100.0% | 100.0% | 0.769 |

**Table S5:** Predictive performance of the TabPFN model (coagulation indicators excluded)

| **Dataset** | **Overall Accuracy** | | **ROC AUC  (95% CI)** | **Sensitivity** | **Specificity** | **Precision** | **F1  Score** |
| --- | --- | --- | --- | --- | --- | --- | --- |
| **Training** | 95.3% | 0.999 (0.998 - 1.000) | | 93.8% | 96.8% | 96.5% | 0.951 |
| **Internal Testing** | 84.8% | 0.945 (0.900 - 0.981) | | 86.0% | 83.6% | 82.7% | 0.843 |
| **Temporal  Validation** | 81.4% | 0.914 (0.830 - 0.971) | | 100.0% | 65.6% | 71.05% | 0.832 |
| **External  Validation** | 70.5% | 0.850 (0.759 - 0.929) | | 88.6% | 55.8% | 62.0% | 0.729 |

**Figure S1:** Detailed performance evaluation of the TabPFN foundation model across all study cohorts. (A) Decision Curve Analysis (DCA) illustrating the clinical net benefit of the model in the retrospective training cohort. The blue line represents the TabPFN model, the red dashed line represents the assumption that all patients have CVA, and the green dashed line represents the assumption that no patients have CVA. (B–E) Confusion matrices detailing the classification results across the four study cohorts: the retrospective training cohort (B, N=428), retrospective internal testing cohort (C, N=105), temporal validation cohort (D, N=59), and external validation cohort (E, N=78). The matrices display the absolute number of True Negatives (top-left), False Positives (top-right), False Negatives (bottom-left), and True Positives (bottom-right). 0: Non-CVA (Control group); 1: Cough Variant Asthma (Case group).

**Figure S2:** Calibration curves of the TabPFN and XGBoost models across validation cohorts. (A) Calibration curves for TabPFN after post-hoc isotonic calibration. (B) Calibration curves for XGBoost based on the original model probabilities without post-hoc recalibration. The dotted diagonal line represents perfect calibration. Curves are shown for the internal testing, temporal validation, and external validation cohorts.
