# Supplementary material for: Screening for Cough Variant Asthma Using the TabPFN Model on Routine Laboratory Data in a Multicenter Cohort: Prospective Validation Study
Source: JMIR Med Inform. 2026 Oct 23;14:e92211. doi: 10.2196/92211 (PMC13743963; doi:10.2196/92211)
Supplement: Supplementary file 2 [file medinform_v14i1e92211_app2.docx]

**Multimedia Appendix 8**

**XGBoost Model Training and Evaluation Results**

**Reproducibility and uncertainty estimation.** The global random seed was fixed at 42. XGBoost hyperparameters were optimized using grid search with stratified 5-fold cross-validation and ROC-AUC as the optimization metric. Cross-validation was used for hyperparameter selection only. The 95% confidence intervals reported for final model performance were estimated using 2,000 bootstrap resamples within each cohort and represent sampling uncertainty. For consistency with Table 2 of the revised manuscript, confidence-interval limits below are displayed using the same rounding precision as the manuscript.

# 1. Complete Hyperparameter Search Grid and Final Selected Parameters

| **Hyperparameter** | **Search grid** | **Final selected value** |
| --- | --- | --- |
| max_depth | 3, 5 | 5 |
| learning_rate | 0.01, 0.05 | 0.05 |
| n_estimators | 30, 60 | 60 |
| reg_alpha | 0.5, 2.0 | 0.5 |
| reg_lambda | 1.0, 10.0 | 1.0 |
| subsample | 0.7, 0.9 | 0.7 |
| colsample_bytree | 0.7, 0.9 | 0.9 |

Hyperparameters were optimized using GridSearchCV with stratified 5-fold cross-validation (shuffle=True), ROC-AUC scoring, and random_state=42.

# 2. Cross-Validation Results for Hyperparameter Selection

| **Item** | **Result** |
| --- | --- |
| Fold AUC scores | 0.93452381, 0.88636364, 0.90963203, 0.90133038, 0.84035477 |
| Mean AUC | 0.894441 |
| Standard deviation | 0.031226 |
| Variance | 0.00097509 |

# 3. Final Model Performance

## Training Set (N=428)

| **Metric** | **Estimate** |
| --- | --- |
| Accuracy | 0.983645 (95% CI 0.970-0.993) |
| Precision | 0.976303 (95% CI 0.954-0.993) |
| Recall | 0.990385 (95% CI 0.975-1.000) |
| Sensitivity | 0.990385 (95% CI 0.975-1.000) |
| Specificity | 0.977273 (95% CI 0.954-1.000) |
| F1 score | 0.983294 (95% CI 0.970-0.993) |
| ROC-AUC | 0.999388 (95% CI 0.997-1.000) |

## Internal Testing Set (N=105)

| **Metric** | **Estimate** |
| --- | --- |
| Accuracy | 0.780952 (95% CI 0.695-0.857) |
| Precision | 0.745455 (95% CI 0.629-0.851) |
| Recall | 0.820000 (95% CI 0.714-0.922) |
| Sensitivity | 0.820000 (95% CI 0.714-0.922) |
| Specificity | 0.745455 (95% CI 0.621-0.852) |
| F1 score | 0.780952 (95% CI 0.689-0.862) |
| ROC-AUC | 0.890182 (95% CI 0.825-0.956) |

## Temporal Validation Set (N=59)

| **Metric** | **Estimate** |
| --- | --- |
| Accuracy | 0.728814 (95% CI 0.610-0.831) |
| Precision | 0.648649 (95% CI 0.488-0.794) |
| Recall | 0.888889 (95% CI 0.759-1.000) |
| Sensitivity | 0.888889 (95% CI 0.759-1.000) |
| Specificity | 0.593750 (95% CI 0.421-0.759) |
| F1 score | 0.750000 (95% CI 0.618-0.853) |
| ROC-AUC | 0.846065 (95% CI 0.742-0.950) |

## External Validation Set (N=78)

| **Metric** | **Estimate** |
| --- | --- |
| Accuracy | 0.756410 (95% CI 0.667-0.846) |
| Precision | 0.666667 (95% CI 0.524-0.791) |
| Recall | 0.914286 (95% CI 0.818-1.000) |
| Sensitivity | 0.914286 (95% CI 0.818-1.000) |
| Specificity | 0.627907 (95% CI 0.487-0.763) |
| F1 score | 0.771084 (95% CI 0.658-0.860) |
| ROC-AUC | 0.911628 (95% CI 0.842-0.982) |

# 4. Computational Efficiency / Time Performance

| **Measure** | **Result** |
| --- | --- |
| Total grid-search time | 10.09 seconds |
| Final model training time | 0.1135 seconds |
| Batch prediction time (105 samples) | 0.0336 seconds |
| Average time per sample (batch prediction) | 0.3197 milliseconds |
| Single-sample inference (average of 100 runs) | 31.0486 milliseconds |
| Model file size | 0.10 MB |
| Processed samples per second | 32 |

**Note.** The cross-validation statistics above describe performance during hyperparameter selection and were not used to derive the confidence intervals reported for the final cohort-level performance estimates.
